# Additive antitumour effect of the epidermal growth factor receptor tyrosine kinase inhibitor gefitinib (Iressa, ZD1839) and the antioestrogen fulvestrant (Faslodex, ICI 182,780) in breast cancer cells

**DOI:** 10.1038/sj.bjc.6601504

**Published:** 2004-01-06

**Authors:** S Okubo, J Kurebayashi, T Otsuki, Y Yamamoto, K Tanaka, H Sonoo

**Affiliations:** 1 Department of Breast and Thyroid Surgery, Kawasaki Medical School, 577 Matsushima, Kurashiki, Okayama 701-0192, Japan; 2 Department of Hygiene, Kawasaki Medical School, 577 Matsushima, Kurashiki, Okayama 701-0192, Japan

**Keywords:** gefitinib, fulvestrant, breast cancer, HER1, p21, Bcl-2

## Abstract

A high expression level of epidermal growth factor receptor (EGFR)/HER1 has been suggested to lead to a shorter survival time and resistance to endocrine therapy in patients with breast cancer. To test the hypothesis that inhibition of the EGFR signalling pathway affects the antitumour effect of endocrine therapy, an EGFR tyrosine kinase inhibitor (EGFR-TKI), gefitinib, and an oestrogen receptor (ER) antagonist, fulvestrant, were administered to human breast cancer cells. A total of five human breast cancer cell lines were used. The effects of single or combined treatments with gefitinib and/or fulvestrant on cell growth, cell cycle progression and apoptosis were analysed. Changes in the expression levels of cyclin-dependent kinase inhibitors, p21 and p27, an antiapoptotic factor, Bcl-2, and a proapoptotic factor, Bax, were also investigated. All cell lines tested were sensitive to gefitinib (50% growth inhibitory concentration, 10–28.5 *μ*M). Breast cancer cell lines with a high expression level of HER1 or HER2 were more sensitive to gefitinib than the others. Gefitinib induced a significant G1–S blockade in ER-positive KPL-3C cells. Gefitinib induced significant apoptosis in HER1-overexpressing MDA-MB-231 cells. Gefitinib additively increased the antitumour effect of fulvestrant in all three ER-positive cell lines in a medium supplemented with 17*β*-oestradiol. The combined treatment promoted cell cycle retardation in KPL-3C cells, which is associated with an upregulation of p21 by fulvestrant and gefitinib, respectively. Apoptosis was associated with downregulation of Bcl-2 by gefitinib in MDA-MB-231 cells. These results suggest an additive interaction between the EGFR-TKI gefitinib and the antioestrogen fulvestrant in ER-positive breast cancer cells.

Epidermal growth factor receptor (EGFR)/HER1 is a member of the HER/erbB family of transmembrane tyrosine kinases. Binding of specific ligands, such as EGF or transforming growth factor-*α*, to the extracellular domain of EGFR/HER1 results in homodimerisation or heterodimerisation with another member of the HER/erbB family, HER2/erbB2, HER3/erbB3 or HER4/erbB4. This dimerisation induces autophosphorylation of tyrosine residues of the intracellular domain of EGFR/HER1, which activates intracellular signalling cascades such as the Ras/Raf/ERK/MAPK, PI-3K/AKT and PLC*γ* pathways. These interactions modulate several cellular functions such as proliferative activity and apoptosis. Under normal conditions, EGFR/HER1 signalling is tightly controlled by various physiological mechanisms (Yarden and Sliwkowski, 2001).

Aberrant EGFR/HER1 signalling, such as high expression of EGFR/HER1, is causally associated with enhanced tumour cell proliferation and shorter survival in patients with solid tumours such as breast cancer (Arteaga, 2002). It has been reported that EGFR/HER1 is expressed at high levels in at least 20% of breast cancers. This high expression has been suggested to correlate with a shorter survival time and resistance to endocrine therapy in patients with breast cancer (Sainsbury *et al*, 1987; Nicholson *et al*, 1989; Newby *et al*, 1995). In addition, a series of experimental and clinical findings have suggested that aberrant activation of tyrosine receptor kinases, such as EGFR/HER1 and HER2 pathways, play a causal role in the development of antioestrogen resistance in breast cancer (van Agthoven *et al*, 1992; Benz *et al*, 1993; Houston *et al*, 1999; Dowsett *et al*, 2001; Wakeling *et al*, 2001).

Gefitinib (Iressa, ZD1839) is an orally active EGFR-tyrosine kinase inhibitor (TKI) that blocks signal transduction pathways implicated in proliferation and survival of cancer cells (Baselga and Averbuch, 2000). Recent studies have suggested that gefitinib induces cell cycle retardation and apoptosis, and inhibits the growth of several types of human cancer cells expressing EGFR both *in vitro* and *in vivo* (Ciardiello *et al*, 2000; Sirotnak *et al*, 2000; Ciardiello *et al*, 2001; Moasser *et al*, 2001; Moulder *et al*, 2001; Bianco *et al*, 2002; Ciardiello *et al*, 2002; Fujimura *et al*, 2002; Huang *et al*, 2002; Janne *et al*, 2002; Magne *et al*, 2002; Williams *et al*, 2002). In addition, human breast cancer cells overexpressing HER2 or acquiring resistance to the oestrogen receptor (ER) antagonist, fulvestrant (Faslodex, ICI 182,780), have been reported to be particularly sensitive to gefitinib (McClelland *et al*, 2001; Moulder *et al*, 2001). Furthermore, phase I trials of gefitinib in patients with solid tumours refractory to standard chemotherapies have shown good tolerability and promising antitumour activity (Baselga *et al*, 2002; Herbst *et al*, 2002; Ranson *et al*, 2002). These findings suggest that gefitinib will be clinically useful in the treatment of patients with hormone-refractory breast cancer.

To test the hypothesis that inhibition of the EGFR/HER1 signalling pathway affects the antitumour effect of endocrine therapy, gefitinib and fulvestrant were administered to human breast cancer cells. We found that gefitinib increases the antiproliferative effect of fulvestrant in ER-positive breast cancer cells under 17*β*-oestradiol (E2)-supplemented conditions. In addition, this effect is caused by cell cycle retardation (G1–S blockade) and an increase in apoptosis. To investigate more detailed mechanisms of action of gefitinib and fulvestrant, changes in the expression levels of cyclin-dependent kinase inhibitors (CDKIs), p21 and p27, an antiapoptotic protein, Bcl-2 and a proapoptotic protein, Bax, were also investigated.

## MATERIALS AND METHODS

### Reagents

E2 was purchased from Sigma Chemical Co. (St Louis, MI, USA). Both fulvestrant and gefitinib were kindly provided by AstraZeneca (Macclesfield, UK). E2 and fulvestrant were dissolved with 100% ethanol and added to the medium at a final ethanol concentration of 0.2%. Gefitinib was dissolved with DMSO and added to the medium at a final concentration of 0.1%.

### Cell lines

KPL-1, KPL-3C and KPL-4 human breast cancer cell lines were established in our laboratory (Kurebayashi *et al*, 1995; Kurebayashi *et al*, 1996; Kurebayashi *et al*, 1999). KPL-1 cells are derived from a postmenopausal patient with hormone-refractory breast cancer. This cell line is ER*α*-positive and oestrogen responsive *in vitro* but not *in vivo* (Kurebayashi *et al*, 2000a). KPL-3C cells are derived from a premenopausal patient with breast cancer associated with humoral hypercalcemia. This cell line is ER*α*-positive and oestrogen responsive both *in vitro* and *in vivo*. KPL-4 cells are derived from a postmenopausal patient with inflammatory skin metastasis from breast cancer. This cell line expresses a high level of HER2 associated with HER2/*neu* gene amplification. T47D and MDA-MB-231 cell lines were kindly provided by Dr Robert B Dickson (Lombardi Cancer Research Center, Georgetown University Medical Center, Washington DC, USA). All cell lines were routinely cultured in Dulbecco's minimal essential medium (D-MEM) supplemented with 5% fetal bovine serum (FBS).

### Expression levels of ER, progesterone receptor (PgR) and HER family members

The expression levels of ER and PgR in the cell pellets of the breast cancer cell lines were measured by an enzyme immunoassay using the ER-EIA and PgR-EIA kits (Dinabot Inc., Tokyo, Japan), according to the manufacturer's recommendations. The expression levels of HER family members were measured by the multiplex reverse transcription–polymerase chain reaction (RT–PCR) as described previously (Kunisue *et al*, 2000; Kurebayashi *et al*, 2000b). Oligonucleotide primers were designed using a published sequence of each target gene and synthesised by the solid-phase triester method. To amplify both the internal control gene (*β*-actin) and one of the target genes in a single reaction, multiplex PCR was carried out. The relative expression levels were calculated as the density of the product of the respective target genes divided by that of the control gene. In addition, protein expression levels of HER1 and HER2 were measured by Western blotting using anti-HER1 goat polyclonal antibody (Santa Cruz Biotechnology, Santa Cruz, CA, USA) and anti-HER2 mouse monoclonal antibody (Santa Cruz Biotechnology), respectively. Detailed procedure of immunoblot analysis is described below.

### Growth assay and cell cycle analysis

To reduce endogenous oestrogen-like activity, phenol red-free RPMI-1640 medium (Gibco BRL, Bethesda, MD, USA) supplemented with 5% dextran-coated charcoal-stripped FBS (Hyclone, UT, USA) (oestrogen-deprived medium) was used. Approximately 2 × 10^5^ cells well^−1^ were inoculated into 12-well plates (Costar Corning Inc., Corning, NY, USA) and cultured in D-MEM supplemented with 5% FBS for 2 days. Then, the cells were washed twice with phosphate-buffered saline (PBS) and cultured for 2 days in the oestrogen-deprived medium with vehicle (control), 1 nM E2, the indicated concentrations of gefitinib and/or fulvestrant. After cell dispersion with 0.05% trypsin (Difco, Detroit, MI, USA) and 0.02% EDTA in PBS for 10 min, the cell numbers were measured with a Coulter counter (Coulter Electronics Ltd, Harpenden, UK). Triplicate wells were treated in each experiment.

To investigate the effect of the agents on cell cycle progression, a part of the harvested cells were stained with propidium iodide using the CycleTest Plus DNA Reagent kit (Becton Dickinson, San Jose, CA, USA). Flow cytometry was performed with a FACSCaliber flow cytometer (Becton Dickinson), and the DNA histogram was analysed by a CELLQuest version 1.2.2 (Becton Dickinson) as described previously (Kunisue *et al*, 2000).

### Apoptosis assay

Approximately 5 × 10^5^ cells well^−1^ were plated into T-25 flasks (Corning Japan, Tokyo, Japan) and cultured in D-MEM supplemented with 5% FBS for 2 days. Then, the cells were washed twice with PBS and cultured for 2 or 4 days in the oestrogen-deprived medium with vehicle (control), 10 *μ*M gefitinib, 1 nM E2, 1 nM E2 plus 100 nM fulvestrant, 1 nM E2 plus 10 *μ*M gefitinib or 1 nM E2 plus 100 nM fulvestrant and 10 *μ*M gefitinib. Duplicate flasks were trypsinised and harvested. The percentages of apoptotic cells were measured with a FACSCaliber flow cytometry (Becton Dickinson) using an *in situ* cell death detection kit (Roche Diagnostics GmbH, Mannheim, Germany), according to the manufacturer's recommendations as described previously (Kunisue *et al*, 2000).

### Immunoblot analysis

Approximately 4 × 10^5^ cells well^−1^ were plated in six-well plates (Costar Corning Inc.) and cultured in D-MEM supplemented with 5% FBS for 2 days. Then, the cells were washed twice with PBS and cultured for 2 days in the oestrogen-deprived medium with vehicle (control), 10 *μ*M gefitinib, 1 nM E2, 1 nM E2 plus 100 nM fulvestrant, 1 nM E2 plus 10 *μ*M gefitinib or 1 nM E2 plus 100 nM fulvestrant and 10 *μ*M gefitinib. Then, the cells were washed once with cold PBS and lysed by 200 *μ*l well^−1^ of cold RIPA buffer (50 mM Tris-HCl (pH 7.4), 150 mM NaCl, 1% Nonidet P-40, 1% deoxycholic acid sodium, 0.05% SDS) plus 4 *μ*l well^−1^ of protease inhibitor (Sigma Chemical Co.). The cell lysate was clarified by centrifugation at 14 000 **g** at 4°C for 10 min. In total, 20–30 *μ*g protein was heated in Laemmli gel loading buffer for 5 min at 95°C and subjected to electrophoresis on 12.5% polyacrylamide gel (Bio-Rad, Richmond, CA, USA). The protein was transferred to nitrocellulose membranes (Amersham Life Sciences, Buckinghamshire, UK) and immunoblotted with appropriate primary antibodies. For detection, the blots were incubated with the appropriate secondary antibodies conjugated with horseradish peroxidase (Santa Cruz Biotechnology), and developed using ECL Plus Western Blotting Detection Reagents (Amersham Life Sciences), according to the instructions of the manufacturer. Both anti-p21 and anti-p-27 monoclonal antibodies were obtained from Nippon Becton Dickinson (Tokyo, Japan). Anti-Bcl-2 and anti-BAX monoclonal antibodies were obtained from Dako Japan (Tokyo, Japan) and Medical and Biological Laboratories Co. (Tokyo, Japan), respectively. Both anti-actin and anti-*α*-tubulin monoclonal antibodies were obtained from Santa Cruz Biotechnology. Densinometry was performed using Image-Pro Plus Version 4.0 (Planetron, Tokyo, Japan). The intensities of the proteins were normalised to the actin or *α*-tubulin band and quantified by comparing with those of control cells. Reproducibility was confirmed in at least two separate experiments.

### Statistical analysis

All values are expressed as the mean±s.e. ANOVA analysis with StatView computer software (ATMS Co., Tokyo, Japan) was used to compare the differences between two groups. A two-sided *P*-value less than 0.05 was considered to be statistically significant.

## RESULTS

### Expression levels of ER, PgR and HER family members

KPL-1 and KPL-3C cells expressed a high level of ER and undetectable PgR in the oestrogen-deprived condition (but PgR is inducible by an E2 supplementation) (Kurebayashi *et al*, 1996; Kurebayashi *et al*, 2000a). T47D cells expressed a low level of ER associated with a very high level of PgR in the oestrogen-deprived condition. Both KPL-4 and MDA-MB-231 cells expressed neither ER nor PgR (Kurebayashi *et al*, 2000b; Table 1

**Table 1:** Expression levels of ER, PgR and HER family members in human breast cancer cell lines tested

**Cell line**	**ER^a^**	**PgR^a^**	**HER1^b^**	**HER2^b^**	**HER3^b^**	**HER4^b^**
KPL-1	79.7±13.3	ND^c^	1.5	3.0	2.5	1.0
KPL-3C	90.9±18.2	ND	0.8	3.0	2.7	1.3
T47D	32.5±1.0	8480.0±720.0	1.3	3.7	2.0	2.2
KPL-4	ND	ND	1.8	9.5	2.0	1.1
MDA-MB-231	ND	ND	3.0	2.6	0.8	1.1

a Femtomoles mg^−1^ protein measured by EIA (mean±s.e.).

b Expression ratio in comparison with internal control (*β*-actin) by multiplex RT–PCR.

c Not detectable.

). In contrast, all cell lines expressed a detectable level of each HER family member by the RT–PCR. KPL-4 cells expressed a highest level of HER2, and MDA-MB-231 expressed a highest level of HER1 (Table 1). Western blot analysis also supported these findings (Figure 1

**Figure 1:**
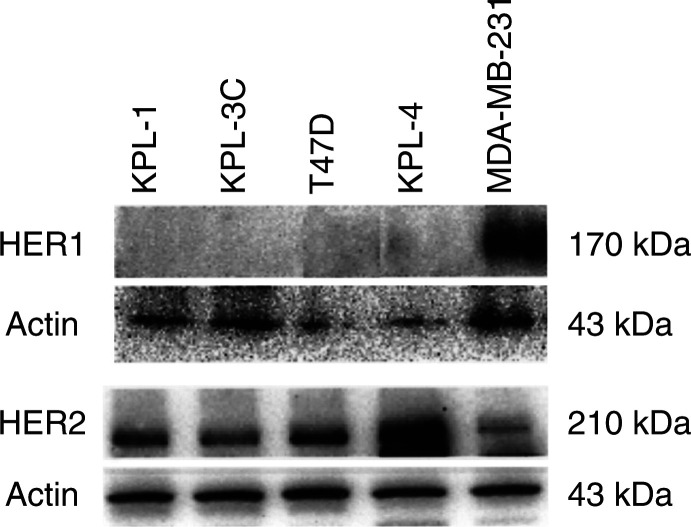
Western blot analysis on expression levels of EGFR/HER1 and HER2 in five breast cancer cell lines tested. The harvested cells were lysed, and the lysate was subjected to electrophoresis on polyacrylamide gel. The protein was transferred to nitrocellulose membranes and immunoblotted with appropriate primary antibodies. For detection, the blots were incubated with the appropriate secondary antibodies conjugated with horseradish peroxidase, and developed using ECL Plus Western Blotting Detection Reagents. Actin was used as the internal control.

).

### Antitumour effect of gefitinib alone

**Figure 2:**
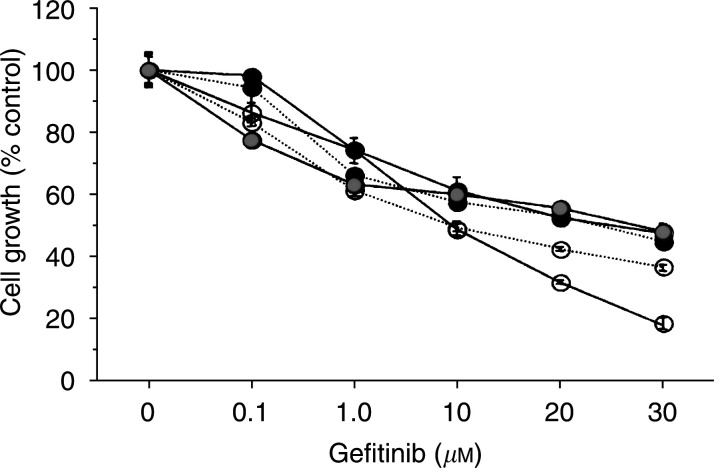
Effect of gefitinib alone on the cell growth of five human breast cancer cell lines tested. Semiconfluent cells were cultured for 2 days in the oestrogen-deprived medium with vehicle, 0.1, 1.0, 10, 20 or 30 *μ*M gefitinib. After cell dispersion, the cell numbers were measured with a Coulter counter. Triplicate wells were treated in each experiment. Values represent means±s.e. 
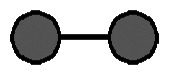
, KPL-1; •- - -•, KPL-3C; 
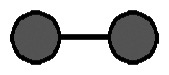
, T47D; ○- - -○, KPL-4; and ○—○, MDA-MB-231.

Gefitinib alone dose dependently inhibited the growth of all breast cancer cell lines tested (Figure 2

). The mean 50%-growth inhibitory concentrations of gefitinib were 28.5±1.5 *μ*M for KPL-1 cells, 25.0±1.0 *μ*M for KPL-3C cells, 25.0±3.0 *μ*M for T47 D cells, 10.0±1.0 *μ*M for KPL-4 cells and 10.0±0.5 *μ*M for MDA-MB-231 cells, respectively (*n*=2 each). HER1-overexpressing MDA-MB-231 and HER2-overexpressing KPL-4 cell lines were more sensitive to gefitinib than the other cell lines.

### Effects of E2, fulvestrant and gefitinib on cell growth

**Figure 3:**
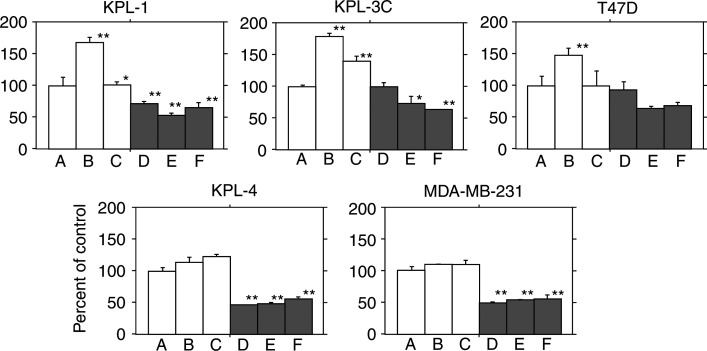
Effect of E2, fulvestrant and gefitinib on the cell growth of five human breast cancer cell lines tested. Semiconfluent cells were cultured for 2 days in the oestrogen-deprived medium with vehicle (control, A), 1 nM E2 (B), 1 nM E2 plus 100 nM fulvestrant (C), 1 nM E2 plus 10 *μ*M gefitinib (D), 1 nM E2 plus 100 nM fulvestrant and 10 *μ*M gefitinib (E) or 10 *μ*M gefitinib alone (F). After cell dispersion, the cell numbers were measured with a Coulter counter. Triplicate wells were treated in each experiment. Values represent means±s.e. ^*^P<0.05 and ^**^P<0.01 in comparison with control.

In KPL-1, KPL-3C and T47D cells, 1 nM E2 significantly stimulated their growth. Both 100 nM fulvestrant and 10 *μ*M gefitinib significantly reduced the growth-stimulatory effect of E2. Simultaneous addition of fulvestrant and gefitinib additively reduced the growth-stimulatory effect of E2. In either KPL-4 or MDA-MB-231 cells, gefitinib significantly inhibited the cell growth but fulvestrant did not (Figure 3

). In contrast, no significant growth inhibitory effect of fulvestrant was observed in all cell lines tested, and the simultaneous addition of fulvestrant and gefitinib did not additively inhibit the growth of the ER-positive cell lines in the absence of E2 (data not shown).

**Figure 4:**
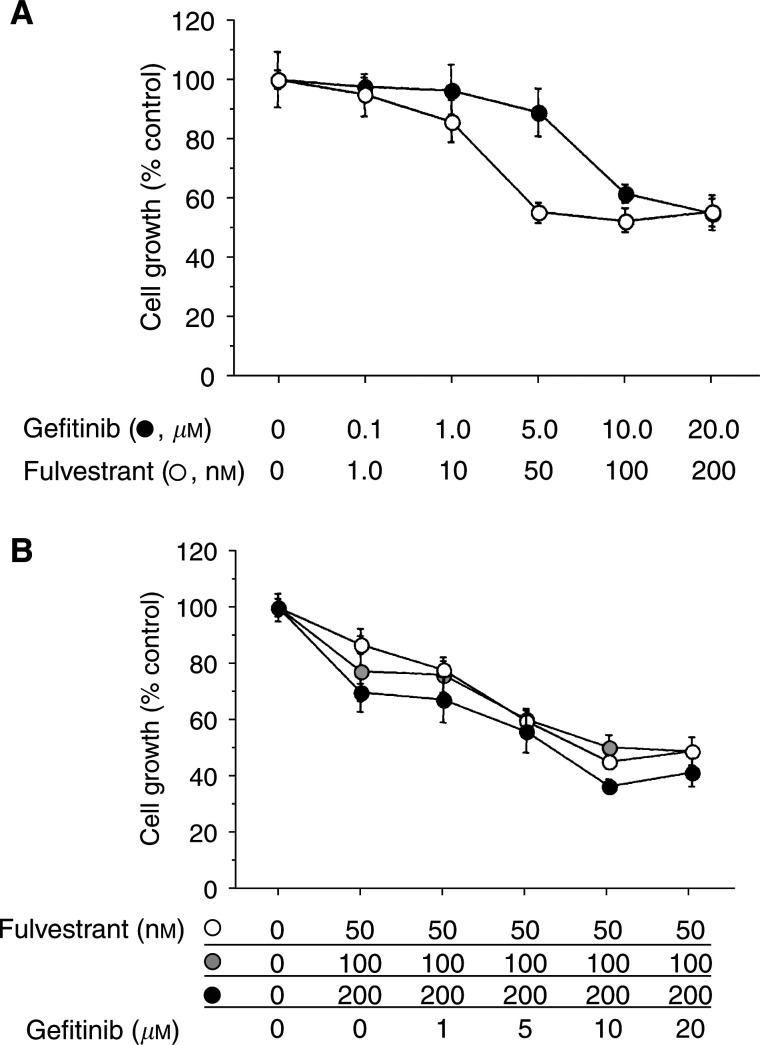
Effect of E2, fulvestrant and gefitinib on the cell growth of ER-positive KPL-3C cells. Semiconfluent cells were cultured for 2 days in the oestrogen-deprived medium with 1 nM E2 plus the indicated concentrations of gefitinib or ICI 182,780 (**A**). To clarify additive antitumour effect of gefitinib and ICI 182,780, semiconfluent cells were cultured for 2 days in the oestrogen-deprived medium with 1 nM E2, 50, 100 or 200 nM ICI 182,780 plus the indicated concentrations of gefitinib (**B**). After cell dispersion, the cell numbers were measured with a Coulter counter. Triplicate wells were treated in each experiment. Values represent means±s.e.

To clarify the additive antitumour effect of gefitinib and fulvestrant, various concentrations of gefitinib and fulvestrant were administered to KPL-3C cells under E2-supplemented conditions. Either gefitinib or fulvestrant dose dependently inhibited the growth of KPL-3C cells (Figure 4A

). Gefitinib (0.1–20 *μ*M) additively increased the antitumour activity of fulvestrant (1–200 nM) in KPL-3C cells (Figure 4B).

### Effects of E2, fulvestrant and gefitinib on cell cycle progression

In KPL-3C cells, 1 nM E2 significantly increased an S-phase fraction and decreased a G0/G1-phase fraction, that is, stimulated a G1–S transition. Both 100 nM fulvestrant and 10 *μ*M gefitinib significantly inhibited the G1–S transition stimulated by E2, that is, lead to the G1–S blockade. The simultaneous addition of fulvestrant and gefitinib additively reduced the upregulation of a G1–S transition by E2. A slight increase in a sub-G1 fraction was observed in the cells treated with gefitinib alone. Gefitinib alone significantly increased the sub-G1 fraction in MDA-MB-231 cells (Table 2

**Table 2:** Effects of E2, fulvestrant and gefitinib on cell cycle progression in KPL-3C and MDA-MB-231 cells

**Cell line**	**Treatment**	**G0/G1**	**S**	**G2/M**	**Sub-G1**
KPL-3C	Control	69.0±0.1^a^	15.5±0.5	12.7±0.7	2.9±0.1
	E2	57.7±0.3**	21.6±0.4**	18.4±0.4**	2.3±0.3
	E2+FUL^b^	62.9±0.1**	17.1±0.1*	15.2±0.2**	4.8±0.2**
	E2+GEF^c^	68.1±0.1	13.9±0.1*	14.7±0.3**	3.2±0.2
	E2+FUL+GEF	80.2±0.2**	7.9±0.1**	8.2±0.2**	3.7±0.3
	GEF	79.5±1.5**	7.1±0.8**	7.2±0.2**	5.7±0.2**
					
MDA-MB-231	Control	58.8±0.8	16.7±0.3	14.7±0.3	9.8±0.2
	GEF	49.3±0.7*	16.5±0.5	12.6±0.4*	21.6±0.6**

a Percentages (mean±s.e.).

b Fulvestrant.

c Gefitinib.

* *P*<0.05;

** *P*<0.01 in comparison with the respective control.

).

### Effect of gefitinib on apoptosis

**Figure 5:**
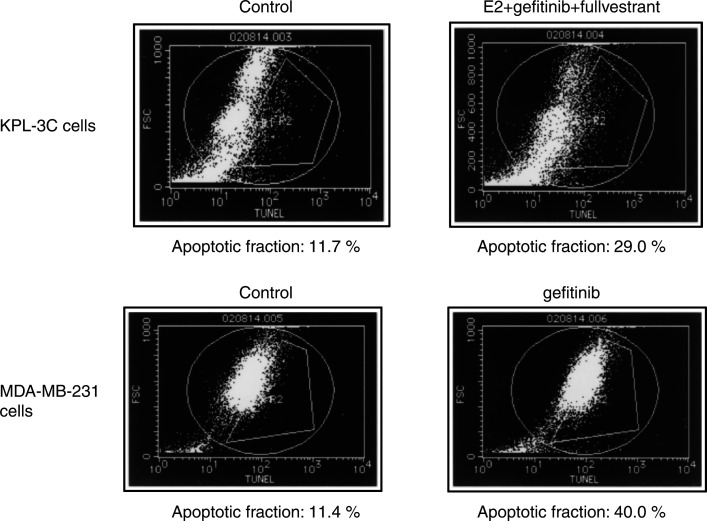
Analyses of apoptosis in KPL-3C and MDA-MB-231 cells treated with E2, fulvestrant and/or gefitinib. Semi-confluent cells were cultured for 4 days in the oestrogen-deprived medium with vehicle (control), 1 nM E2 plus 100 nM fulvestrant and 10 *μ*M gefitinib or 10 *μ*M gefitinib alone. The percentages of apoptotic cells were measured with a flow cytometry using an *in situ* cell death detection kit. Note the increase in apoptotic cells by the addition of fulvestrant and gefitinib to E2 in KPL-3C cells and in MDA-MB-231 cells treated with gefitinib alone.

To clarify whether the sub-G1 fraction detected by the cell cycle analysis represented apoptosis, KPL-3C cells treated with E2, fulvestrant and gefitinib and MDA-MB-231 cells treated gefitinib alone were subjected to the apoptosis assay. As shown in Figure 5

, a significant increase in the apoptotic fraction was observed in the treated cells in comparison with the respective control cells.

### Effects of E2, fulvestrant and gefitinib on expression levels of p21, p27, Bcl-2 and Bax

**Figure 6:**
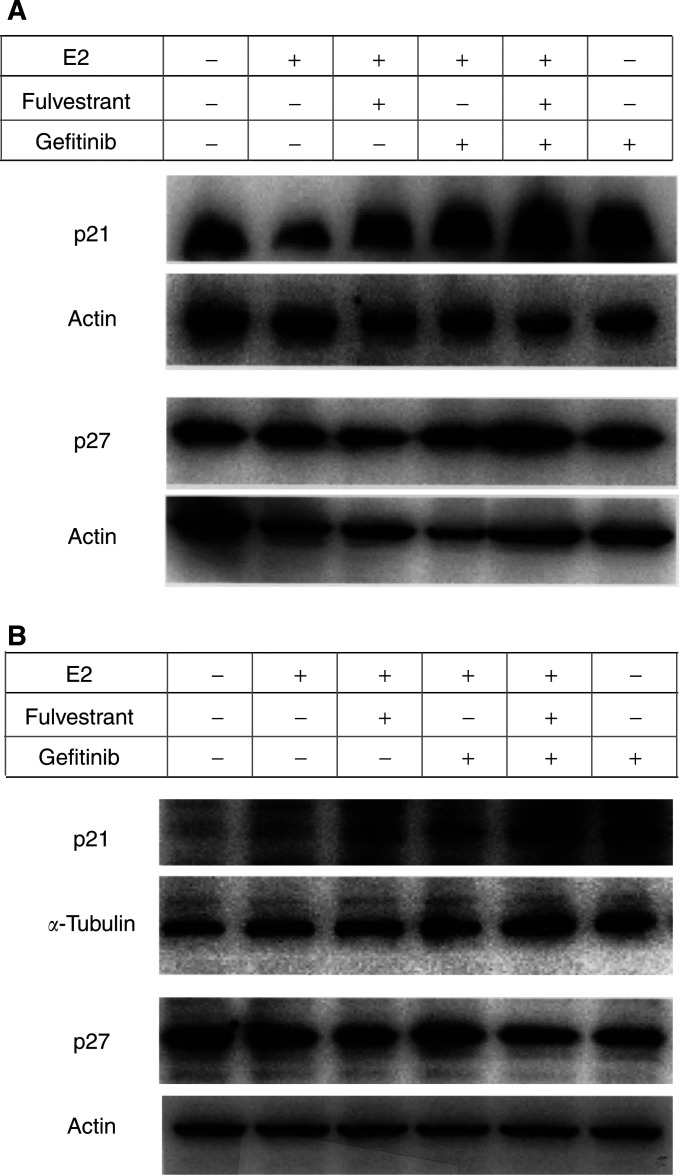
Western blot analysis on expression levels of CDKIs p21 and p27 in KPL-3C (**A**) and MDA-MB-231 (**B**) cells. Semi-confluent cells were cultured for 2 days in the oestrogen-deprived medium with vehicle (control), 1 nM E2, 1 nM E2 plus 100 nM fulvestrant, 1 nM E2 plus 10 *μ*M gefitinib, 1 nM E2 plus 100 nM fulvestrant and 10 *μ*M gefitinib or 10 *μ*M gefitinib alone. The harvested cells were lysed, and the lysate was subjected to electrophoresis on polyacrylamide gel. The protein was transferred to nitrocellulose membranes and immunoblotted with appropriate primary antibodies. For detection, the blots were incubated with the appropriate secondary antibodies conjugated with horseradish peroxidase, and developed using ECL Plus Western Blotting Detection Reagents. Actin or *α*-tubulin was used as the internal control.

E2 decreased p21 expression in KPL-3C cells (a 21±15% reduction in comparison with control, *n*=2). Both fulvestrant and gefitinib significantly increased the p21 expression decreased by E2 (30±8 and 45±5% increases, respectively). The simultaneous addition of the two agents additively increased the p21 expression decreased by E2 (a 88±22% increase). Gefitinib alone also increased p21 expression in comparison with control (a 66±28% increase) (Figure 6A

). In contrast, neither E2 nor fulvestrant changed p21 expression in MDA-MB-231 cells. Gefitinib alone significantly increased p21 expression in comparison with control (a 222±4% increase) (Figure 6B). No significant change in p27 expression was observed in either cell lines treated with these three agents (Figure 6A and B).

**Figure 7:**
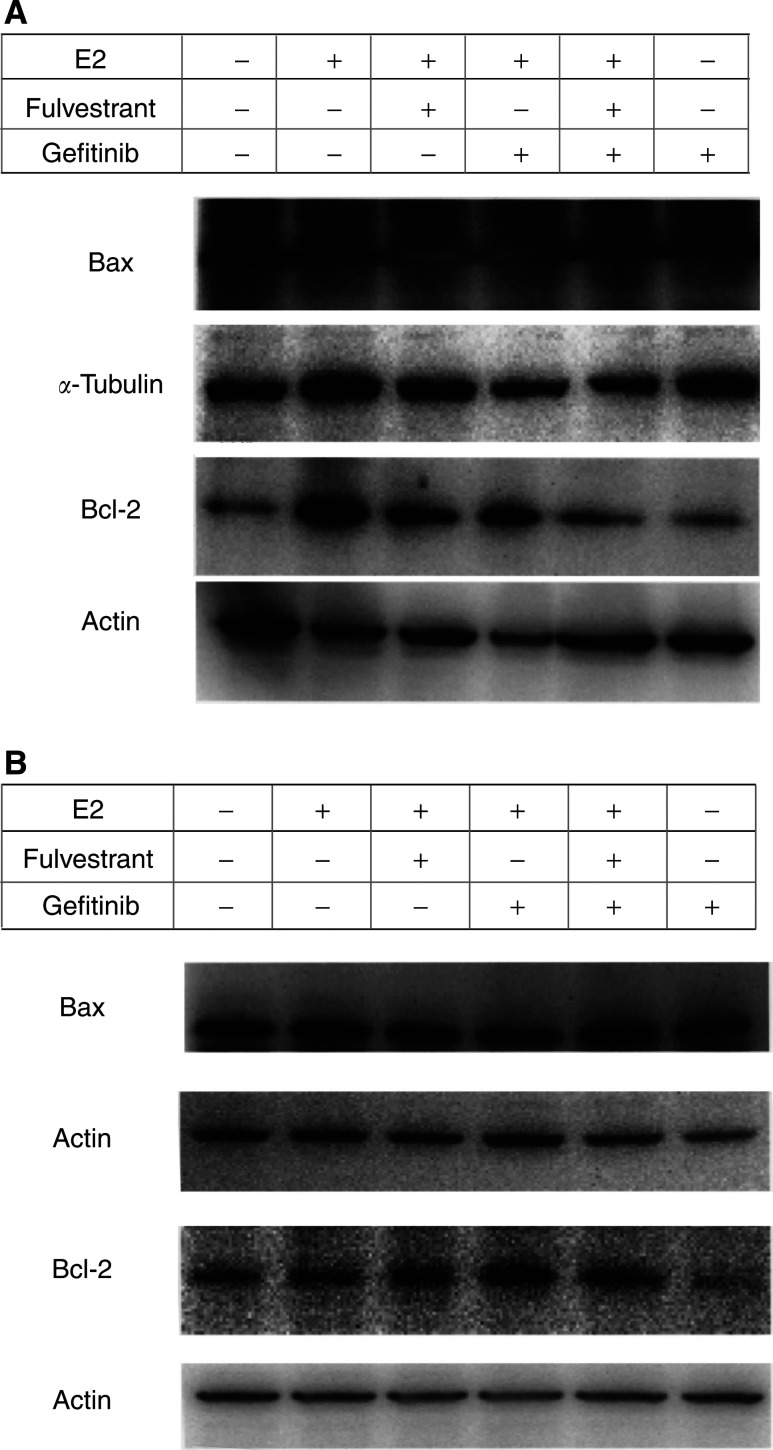
Western blot analysis on the expression levels of a pro-apoptotic protein BAX and an anti-apoptotic protein Bcl-2 in KPL-3C (**A**) and MDA-MB-231 (**B**) cells. Semi-confluent cells were cultured for 2 days in the oestrogen-deprived medium with vehicle (control), 1 nM E2, 1 nM E2 plus 100 nM fulvestrant, 1 nM E2 plus 10 *μ*M gefitinib, 1 nM E2 plus 100 nM fulvestrant and 10 *μ*M gefitinib or 10 *μ*M gefitinib alone. The harvested cells were lysed, and the lysate was subjected to electrophoresis on polyacrylamide gel. The protein was transferred to nitrocellulose membranes and immunoblotted with appropriate primary antibodies. For detection, the blots were incubated with the appropriate secondary antibodies conjugated with horseradish peroxidase, and developed using ECL Plus Western Blotting Detection Reagents. Actin or *α*-tubulin was used as the internal control.

E2 significantly increased Bcl-2 expression in KPL-3C cells (a 196±120% increase). Either fulvestrant or gefitinib decreased the Bcl-2 expression increased by E2 (105±33 and 130±14% reductions, respectively). The simultaneous addition of the two agents additively decreased the Bcl-2 expression increased by E2 (a 169±25% reduction). Gefitinib alone did not significantly decrease Bcl-2 expression (Figure 7A

). In contrast, neither E2 nor fulvestrant changed Bcl-2 expression in MDA-MB-231 cells. Gefitinib alone significantly decreased Bcl-2 expression in comparison with control (a 46±33% reduction) (Figure 7B). No significant change in BAX expression levels was observed in either cell lines treated with these three agents (Figure 7A and B).

**Figure 8:**
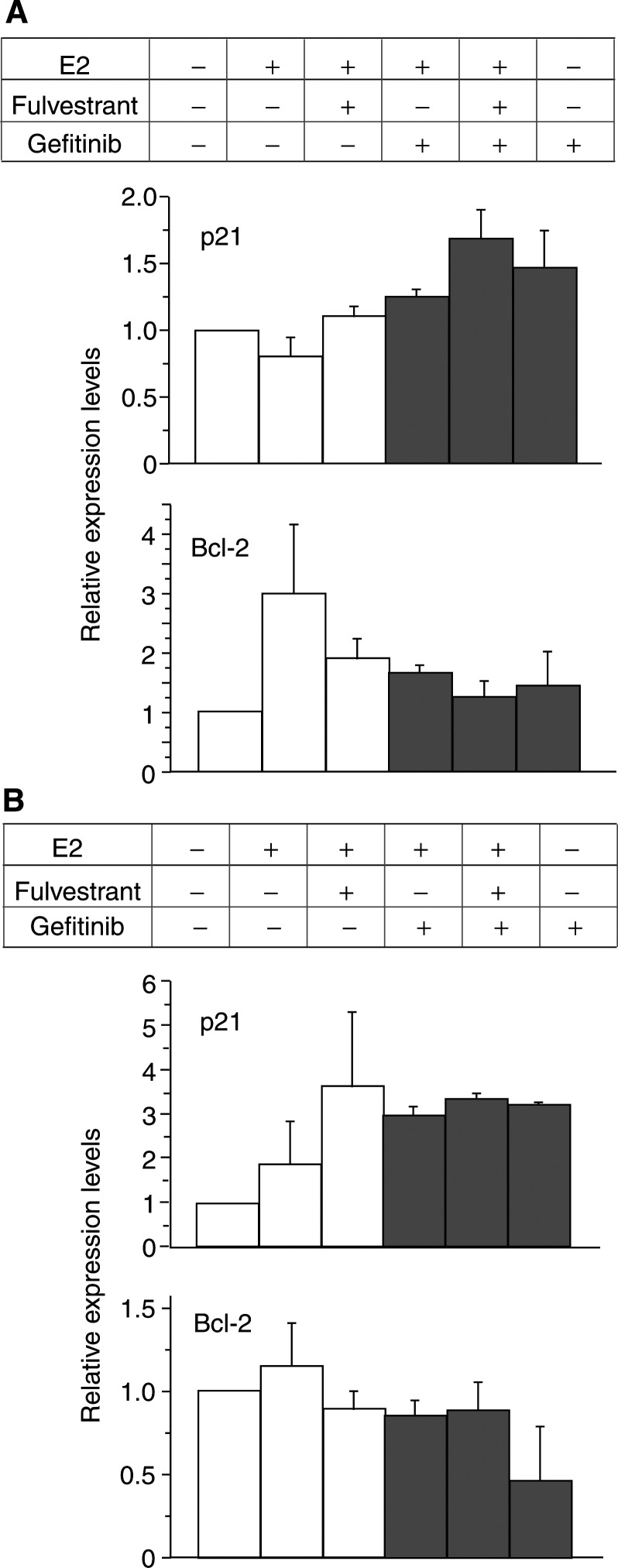
The relative expression levels of p21 and Bcl-2 in KPL-3C (**A**) and MDA-MB-231 (**B**) cells. Western blot bands were normalized to actin or *α*-tubulin bands and quantified by comparing with those of control cells. Data are shown as the means of two separate experiments; bars, s.e.

The relative expression levels of p21 and Bcl-2, which were normalised to actin or *α*-tubulin bands, and quantified by comparing with those of control cells, are shown in Figure 8A and B

.

## DISCUSSION

The mechanism of action of the EGFR-TKI gefitinib has been extensively studied in the last few years. It has been known that gefitinib selectively inhibits ATP binding to the ATP site of EGFR/HER1 and this leads to inhibition of ligand-dependent EGFR autophosphorylation and of transactivation of downstream signalling pathways, such as Ras/Raf/ERK/MAPK and PI3K/AKT cascades. Blockade of the EGFR signalling pathways results in not only retardation of cell cycle progression but also induction of apoptosis in EGFR-expressing tumour cells. It has been suggested that the cell cycle retardation is mediated by an increase in the expression level of a CDKI p27 (Moasser *et al*, 2001; Albanell *et al*, 2002; Janne *et al*, 2002), and that the induction of apoptosis is mediated by a decrease in the expression level of an antiapoptotic protein Bcl-2 (Bianco *et al*, 2002; Ciardiello *et al*, 2002; Nicholson *et al*, 2002) or by activation of a proapoptotic protein BAD (Gilmore *et al*, 2002). Very recently, it has been reported that the EGFR-TKI gefitinib increases both p27 and p21 proteins associated with CDK2–cyclin-E and CDK2–cyclin-A complexes in HER1-overexpressing head and neck squamous carcinoma cells (Di Gennaro *et al*, 2003). In addition, gefitinib has been suggested to decrease the expression levels of proangiogenic factors, such as vascular endothelial growth factor and basic fibroblast growth factor in tumour cells, to inhibit migration and tube formation of endothelial cells and to reduce the invasive potential of tumour cells (Ciardiello *et al*, 2001; Bianco *et al*, 2002; Fujimura *et al*, 2002; Hirata *et al*, 2002). Synergy of these mechanisms of action of gefitinib may provide a drastic antitumour effect *in vivo*.

It has been recognised that activation of oncogenic tyrosine kinase pathways, such as EGFR signaling pathway, leads to acquisition of resistance to radiotherapy and cytotoxic chemotherapy (Skorski, 2002). Actually, several experimental studies have suggested that the combined treatment with gefitinib and ionising radiation or cytotoxic agents, such as taxanes and cisplatin, synergistically inhibit the growth of tumour cells both *in vitro* and *in vivo* (Ciardiello *et al*, 2000; Sirotnak *et al*, 2000; Ciardiello *et al*, 2001; Bianco *et al*, 2002; Ciardiello *et al*, 2002; Huang *et al*, 2002; Magne *et al*, 2002; Williams *et al*, 2002). However, no synergistic or additive activity has been reported with gefitinib and a cytotoxic agent in phase III clinical trials (Giaccone *et al*, 2002; Johnson *et al*, 2002). Furthermore, it has been suggested that high expression of EGFR or HER2 causes the development of acquired endocrine resistance in endocrine-responsive breast cancer (Benz *et al*, 1993; Houston *et al*, 1999). Therefore, it might be possible that gefitinib will enhance the antitumour effect of endocrine agents.

In the present study, gefitinib additively increased the antitumour effect of the ER antagonist fulvestrant in ER-positive human breast cancer cells lines tested (Figures 3 and 4B). Cell cycle and apoptosis analyses revealed that gefitinib and fulvestrant additively induced a G1–S blockade and apoptosis in these cells (Table 2 and Figure 5). In addition, Western blot analysis showed that both agents additively upregulated the expression level of a CDKI p21 decreased by E2 and additively downregulated the expression level of an antiapoptotic protein Bcl-2 increased by E2 (Figures 6, 7 and 8). These findings suggest that the upregulation of p21 expression by these agents may cause the retardation of G1–S transition, and that the downregulation of Bcl-2 expression may cause the induction of apoptosis. Unexpectedly, expression levels of another CDKI p27 did not change during treatment with gefitinib and fulvestrant (Figure 6). To our knowledge, this is the first report suggesting that gefitinib upregulates a CDKI p21 expression decreased by E2 and this may induce, at least in part, the G1–S blockade. In addition, this is also the first report suggesting that gefitinib downregulates the intrinsic Bcl-2 expression increased by E2 and this may cause, at least in part, the induction of apoptosis. However, it is hard to clarify whether these additive effects, including antitumour activity, induction of G1–S blockade, induction of p21 expression and decrease in Bcl-2 expression, produced by gefitinib and fulvestrant in ER-positive breast cancer cells are mediated by the interaction of these drugs or by the summation of two independent actions.

Gefitinib induced neither a significant G1–S blockade nor reduction in S-phase fraction in MDA-MB-231 cells. Otherwise, a significant increase in the p21 expression level was observed (Figures 6B and 8B). On the other hand, a significant downregulation of Bcl-2 and increase in apoptosis was observed at the same time (Table 2 and Figures 7B and 8B). It might be possible that a G1–S blockade and reduction in S-phase fraction induced by gefitinib was drowned by the induction of a massive apoptosis by gefitinib in the cell cycle analysis or a G1–S blockade appeared some time before the induction of apoptosis. Further analyses, such as time course studies of expression levels of CDKIs and apoptosis-modulating proteins are needed to understand in greater detail the mechanism of action of gefitinib.

Clinically, combined treatments with endocrine therapy and an inhibitor of growth factor signalling pathways are thought to be promising. Rationales of this hypothesis are follows: (1) clinical studies have suggested that ER-positive breast cancers with a high expression level of EGFR or HER2 are frequently resistant to endocrine therapy and have a shorter survival time (Sainsbury *et al*, 1987; Nicholson *et al*, 1989; van Agthoven *et al*, 1992; Newby *et al*, 1995; Dowsett *et al*, 2001), (2) artificially increasing expression of EGFR or HER2 to high levels in ER-positive breast cancer cells leads to an antioestrogen-resistant phenotype (Benz *et al*, 1993; Houston *et al*, 1999), (3) a prolonged exposure of an antioestrogen to ER-positive breast cancer cells sometimes results in upregulation of EGFR or HER2 expression and an antioestrogen-resistant phenotype (Long *et al*, 1992; McClelland *et al*, 2001; Nicholson *et al*, 2002), (4) we and others have suggested that an anti-HER2 monoclonal antibody or inhibitor of HER2 signalling pathway enhances an antitumour effect of antioestrogens in ER-positive breast cancer cells with a moderate or high level of HER2 expression (Witters *et al*, 1997; Kunisue *et al*, 2000; Kurokawa *et al*, 2000). Actually, combination therapy with trastuzumab and an endocrine agent, the aromatase inhibitor anastrozole, has been tested in a clinical trial (Winer and Burstein, 2001). Interestingly, it has been reported that a combined treatment with gefitinib and trastuzumab cooperatively inhibits the growth of breast cancer cells (Normanno *et al*, 2002). It might be an attractive approach to use this combination for the treatment of antioestrogen-resistant breast cancer.

In conclusion, the present study has suggested that simultaneous administration of the EGFR-TKI gefitinib and the ER antagonist fulvestrant may be more efficacious than either agent alone for the treatment of patients with ER-positive breast cancer, which express HER1 at various levels. In addition, the additive antitumour effect of these agents may be mediated by the additive G1–S blockade mediated by upregulation of p21 and by the additive induction of apoptosis mediated by downregulation of Bcl-2. These findings should be clarified in preclinical *in vivo* studies and in prospective randomised clinical trials in the near future.
